# Predicting health-related quality of life for patients with gastroesophageal cancer

**DOI:** 10.1007/s11136-025-04097-5

**Published:** 2026-02-03

**Authors:** Steven C. Kuijper, Irene Cara, Gijs Geleijnse, Marije Slingerland, Grard A. P. Nieuwenhuijzen, Sjoerd M. Lagarde, Bastiaan R. Klarenbeek, Ewout A. Kouwenhoven, Richard van Hillegersberg, Rob H. A. Verhoeven, Hanneke W. M. van Laarhoven

**Affiliations:** 1 Department of Medical Oncology, Amsterdam UMC location University of Amsterdam, ZH 8D36, De Boelelaan 1117, 1081 HV Amsterdam, The Netherlands; 2 Cancer Center Amsterdam, Cancer Treatment and Quality of Life, Amsterdam, The Netherlands; 3 Department of Research & Development, Netherlands Comprehensive Cancer Organisation (IKNL), Utrecht, The Netherlands; 4 Department of Medical Oncology, Leiden University Medical center, Leiden, The Netherlands; 5 Department of Surgery, Catharina Hospital, Eindhoven, The Netherlands; 6 Department of Surgery, Erasmus Medical Centre, Rotterdam, The Netherlands; 7 Department of Surgery, Radboudumc, Arnhem, The Netherlands; 8 Department of Surgery, ZGT Hospital, Almelo, The Netherlands; 9 Department of Surgery, University Medical Centre Utrecht, Utrecht, The Netherlands

**Keywords:** Prediction, Health-related quality of life, Gastroesophageal cancer, Elastic-net, Machine learning

## Abstract

**Background:**

Gastroesophageal cancer has a poor prognosis, and treatment significantly impacts health-related quality of life (HRQoL). Accurate prediction of HRQoL changes after treatment can support shared decision-making. This study aimed to develop and validate HRQoL prediction models for patients with gastroesophageal cancer using established risk-prediction models and a newly proposed sequential score model.

**Methods:**

HRQoL data came from the Prospective Observational Cohort Study of Esophageal-Gastric Cancer Patients registry, linked to the Netherlands Cancer Registry. The EORTC QLQ-C30 functioning scales were used as outcomes. Risk-prediction models, based on logistic elastic-net regression, estimated the probability of meaningful HRQoL deterioration at 3, 6, and 12 months post-treatment. The sequential score model, using XGBoost regression, predicted the next HRQoL score at any time. Calibration curves and integrated calibration index (ICI) assessed predictive performance, with Brier scores and AUC for risk-prediction models and root mean squared error plus Out-of-Sample r^²^ for sequential models.

**Results:**

Risk-prediction models showed strong performance (ICI: 0.03–0.08; Brier score: 0.09–0.17; AUC: 0.79–0.87) for predicting significant deterioration in Summary Score, Physical Functioning, and Fatigue, with good calibration. Sequential score models explained up to 40% of the variance in HRQoL scores.

**Conclusion:**

Both models effectively predicted HRQoL in gastroesophageal cancer patients, demonstrating potential to enhance patient care and information sharing through accurate prediction of HRQoL outcomes.

**Supplementary Information:**

The online version contains supplementary material available at 10.1007/s11136-025-04097-5.

## Introduction

It is well established that treatment of gastroesophageal cancer can affect health-related quality of life (HRQoL) both positively as well as negatively [1, 2, 3]. HRQoL is therefore an important factor to consider when patients and caregivers make decisions about treatment strategies. Clinical prediction models have the potential to support caregivers in providing personalized information regarding treatment outcomes, which could help in the process of shared decision making [4, 5].

Successful efforts to predict HRQoL have been made in colorectal cancer, cervical cancer and breast cancer [6, 7, 8, 9]. These models are based on classical statistical models as well as machine learning models to predict HRQoL at a fixed time horizon. However, no such models are currently available for HRQoL prediction for patients with gastroesophageal cancer [10, 11]. Given the high variability of treatment outcomes in terms of survival across patients with this cancer type, a HRQoL prediction model with good predictive performance could be valuable tool in proving treatment-related information [12, 13].

The primary aim of this study was to develop models to predict post-treatment HRQoL at the time of primary diagnosis for patients with gastroesophageal cancer. To achieve this, we employed a well-established modeling methodology that has been successfully used to assess the risk of significant HRQoL deterioration in colorectal cancer patients [7, 8]. Given its strong predictive performance in the colorectal cancer context, this method was deemed an appropriate choice for gastroesophageal cancer data as well. However, while this approach has demonstrated success, it is not without limitations. A key limitation of this approach is that it simplifies the prediction process by focusing exclusively on risk estimation of HRQoL deterioration, potentially overlooking important nuances in how HRQoL evolves over time.

To address this, we introduce a novel modeling methodology in this study—the sequential score model—which aims to predict HRQoL on a continuous time scale. Unlike existing models, this approach offers the potential for more granular and dynamic predictions of HRQoL progression over time. To our knowledge, this method has not yet been applied in this field, but it holds promise as a more flexible and comprehensive tool for estimating HRQoL after treatment onset.

Since HRQoL prediction in gastroesophageal cancer remains a relatively new field, we conducted a comprehensive study to apply and evaluate two different modeling approaches. Our aim is to explore the potential of these models to provide more accurate HRQoL predictions for patients. By offering a quantitative assessment, these models can help patients better understand how their quality of life may be impacted after treatment. This study follows the TRIPOD guidelines, ensuring transparent and rigorous reporting of multivariable prediction models for individual prognosis or diagnosis [14].

## Methods

### Patient population

We used data from 3,305 patients with malignant gastroesophageal cancer who were diagnosed between 2015 and 2021 in the Netherlands and participated in the Prospective Observational Cohort Study of Esophageal-Gastric Cancer Patients (POCOP). The POCOP study was designed to register patients’ self-reported HRQoL before, during and after treatment and was used for this study to obtain data on HRQoL. The complete methodology of POCOP is described elsewhere [15], but in short, individuals involved in the project are directed to project investigators by a member of the medical team. Subsequently, POCOP investigators reach out to potential participants through phone calls and dispatch the questionnaire via mail or email. Once patients provided written informed consent, they are contacted every three months in the initial year, twice in the second year, and annually thereafter, through phone, mail, or email, for the purpose of gathering patient-reported outcome measures.

The POCOP database is routinely linked to the population-based Netherlands Cancer Registry (NCR) in order to obtain patients, tumor and treatment related information. The NCR is a nationwide database that contains records of all individuals diagnosed with a malignant form of cancer. Trained data managers routinely extract details such as diagnosis, tumor stage, and treatment from the electronic medical records of patients, and integrate this information into the NCR. The primary method of identification relies largely on notifications received from the nationwide network and registry of histopathology and cytopathology in the Netherlands (PALGA).

Patients with esophageal cancer (ICD-O-3 topography codes: C15.0–C15.9), gastroesophageal junction/cardia (C16.0), and gastric cancer (C16.1–C16.9) were included in this study. Gastroesophageal junction/cardia tumors were grouped together esophageal cancer.

### Health-related quality of life outcomes

The primary outcome for prediction were outcome scales of the EORTC QLQ-C30 questionnaire: global health status (GHS), physical functioning, role functioning, emotional functioning, social functioning, nausea/vomiting, fatigue, pain, appetite loss, diarrhea, dyspnea, constipation, insomnia and financial difficulties, as well as the summary score of the QLQ-C30 which was calculated as average of the QLQ-C30 outcome scores, excluding global health status and financial difficulties [16, 17, 18]. All items in the QLQ-C30 questionnaire are scored using a Likert scale, and linearly transformed to be scaled between 0 and 100. To promote readability of the manuscript, we presented the results the GHS and Summary Score in the main text given that these outcome reflect general HRQoL. Additionally, we present the best performing subscales in the main text. Results from all other scales can be found in Supplementary Figs. 1–3 and in Supplementary Table 6.

### Predictor variables

For the risk prediction model, we used a total of 78 candidate predictors known at baseline. We used 15 clinical variables from the NCR: sex, age, height, weight, cT, cN, cM, performance status, tumor differentiation grade, hemoglobin (HB), albumin, lactic acid dehydrogenase (LDH), creatinine, Lauren classification, tumor morphology and treatment (modeled as a single categorical covariate). From POCOP, we used 63 variables: the baseline QLQ-C30 scale scores, baseline EORTC QLQ-OG25 symptom scores, smoking cigarette/cigar/pipe, tube feeding, drinking alcohol, education level, married, EuroQoL-5d scales, Worry of Progression Scale (WOPS) scores and Hospital Anxiety and Depression Scale (HADS) scale scores.

In addition to the abovementioned predictors, the responses to previous QLQ-C30 and QLQ-OG25 questionnaires, the time between questionnaires and the time between treatment onset and previous questionnaires were also selected for the sequential score model.

### Model pipeline and development

**Fig. 1:**
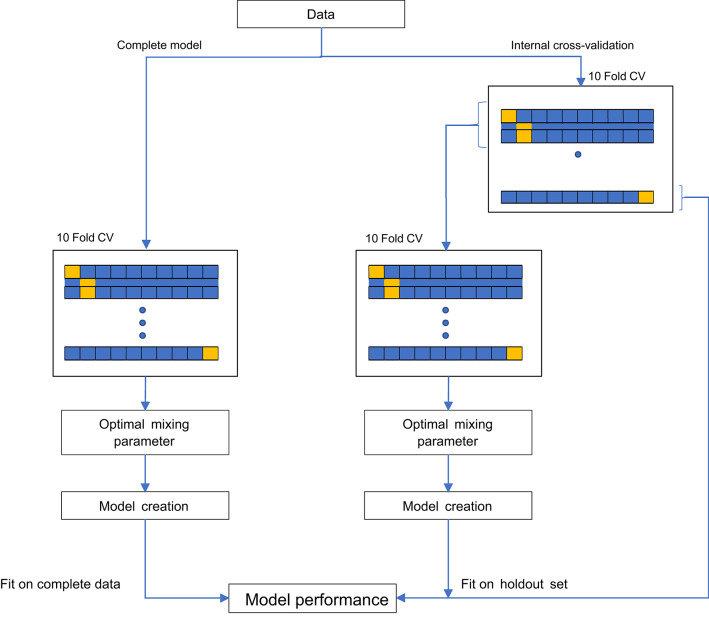
Modeling and validation pipeline for the risk-prediction models and the sequential score prediction models

Two types of models were developed to make HRQoL predictions after treatment onset. Although modeling and prediction of HRQoL was fundamentally different in each approach, the general pipeline and validation was similar (Fig. 1). The modeling details of each approach are described below, but the pipeline can be summarized as follows. First, candidate predictors with more than 50% of missing data were removed from the dataset, which resulted in the removal of tube feeding, smoking cigar, smoking pipe, drinking alcohol, OG25 hair loss symptom scale, and the Worry Of Progression Scale. Any further missing data, reported in Supplementary Table 1, were imputed using a random forest imputation (for the risk-prediction models) or K-Nearest-Neighbors imputation (for the sequential score models), except for the categorical variables tumor morphology, differentiation grade and Lauren classification for which a category “unknown” was used as they were not assumed to be missing at random [19, 20]. Models were trained on the complete dataset with feature selection and their performance was evaluated on the same data (complete model performance). Using 10-fold internal cross-validation, in which all previous steps that were used in model development on the complete dataset were repeated in every fold to prohibit leakage and optimism between train and test set, we tested our methodologies for potential overfitting (internal performance). Finally, both the apparent performance as well as the internal performance were evaluated for all models.

#### Model development: risk prediction

The primary aim of the risk prediction models was predicting the probability of experiencing a clinically meaningful deterioration in each of the sixteen QLQ-C30 outcomes at six months and one year after treatment onset. To this end, we developed a total of 48 prediction models, one for each of the sixteen outcomes for 3 months, 6 months and 12 months after treatment onset.

First, for each patient, we determined the value of the outcome at three, six and twelve months after start of treatment. It was required for each patient that they minimally filled-in two questionnaires, one before start of treatment (with a margin of up to14 days post-treatment) and one questionnaire around the prediction horizon (with a margin of +/- 30 days before and after the time horizon). At these time horizons, we determined if patients experienced a clinically meaningful deteriorations compared to before start of treatment using established cut-off points for small deteriorations (Supplementary Table 2) [21]. In Supplementary Table 3 the prevalence of clinically significant deteriorations at 3, 6 and 12 months is also reported.

Then, for each outcome and time horizon, we trained an elastic net logistic regression model on the complete dataset, which resulted in a total of 48 models. Elastic net regression is a method that shrinks non-informative model coefficients to zero and performs variable selection, while being able to handle high dimensional and colinear data. Given the large number of candidate predictors (78) which may likely be highly colinear, elastic net regression was the most fitting solution for model estimation and variable selection. In training the model on all available data, the lambda parameter and mixing parameter required to perform the elastic net regression were optimized using a nested repeated cross-validation (Fig. 1 and Supplementary Table 4). All modeling steps (including handling of missing data) were repeated in the 10-fold cross-validation to obtain the internal validation performance.

#### Model development: sequential score model

The sequential score model is designed to predict the next QLQ-C30 outcome, at any time after treatment onset. A total of 16 models, one for each QLQ-C30 outcome, were developed using XGBoost, a tree boosting approach broadly known and used in the Data Science community for achieving state-of-the-art performance, thanks to the sequential fitting of trees on residuals [22]. Other key characteristics of XGBoost are the capability of handling high dimensional data with missing input and to capture non-linearities.

For each patient, the values of the outcomes at each questionnaire were determined. The time difference between questionnaires and treatment onset had to be in the range of 30 days to 1000 days. XGBoost regression models were trained, for each outcome, based on all available variables at the time of the previous questionnaire plus the time between questionnaires. Bayesian optimization was deployed to optimize the model parameters (column subsample ratio, training instance subsample ratio, gamma, learning rate, maximum tree depth, minimum child weight, number of estimators, loss function, L1 regularization term and L2 regularization term) in a nested repeated cross-validation [23]. The chosen hyperparameters for each model are reported in Supplementary Table 5. In addition to XGBoost, an Elastic net regression model was trained for comparison. As Elastic net requires data imputation, K-Nearest-Neighbors imputation was used for this purpose, while this step was not required for XGBoost. The modeling strategy (Fig. 1) was repeated in a 10-fold-cross-validation, where folds were selected based on patients rather than on questionnaires to avoid data spilling between the training and the validation sets.

### Model performance

For both modeling approaches, the apparent predictive performance and the internally validated performance was evaluated using calibration curves and the integrated calibration index (ICE). The integrated calibration index (ICI) quantifies the average absolute difference between predicted probabilities and observed outcomes, with lower values indicating better overall calibration of the prediction model. For the risk prediction models, calibration curves were constructed by directly plotting observed outcomes with predicted probabilities and fitting a smooth line using LOESS regression with 95% confidence interval [24]. Calibration curves display the agreement between predicted probabilities and observed event rates across groups, thereby providing a visual assessment of a prediction model’s calibration. For the sequential model, calibration curves were constructed by dividing the predicted score values into 10 quantiles and plotting the mean predicted values versus the mean observed scores. For the risk prediction model, the Brier score was calculated as a measure of the mean squared difference between predicted probabilities and observed outcomes, with lower values indicating higher accuracy. The Area Under the Curve (AUC) for each risk prediction model were measured as the area underneath the receiver operating characteristic curve. For the sequential score model, the Root Mean Square Error (RMSE), representing the square root of the average squared prediction errors, and the Mean Absolute Error (MAE), reflecting the average absolute difference between predicted and observed values, were computed to quantify prediction accuracy. In addition, the out-of-sample r^2^ was calculated to assess the proportion of variance explained by the model on unseen data, thereby evaluating its predictive performance and generalizability [25]. Across all analyses, an alpha level of 0.05 was maintained for statistical significance. R version 4.3.1 and Python version 3.12.1 were used for all analyses. The code to reproduce the results, including the Python and R required packages list, can be found at https://github.com/IKNL/HRQoL-prediction-EGC.

### Predictor importance

For the risk-prediction model, feature importance was determined by the absolute value of the standardized regression coefficient. Larger values corresponded with larger feature importance. Feature importance was determined for the complete models. For the sequential score model, the relative importance of each predictor can be determined by the XGBoost gain and this was evaluated on the complete model.

## Results

### Patient characteristics

In total, 3305 patients were included in the dataset on which the prediction models were developed. Of all patients, 86% were diagnosed with esophageal cancer, of whom 89.2% were diagnosed with non-metastatic disease and 10.8% with metastatic disease (Table 1). For patients with gastric cancer, 85.6% were diagnosed with non-metastatic disease and 14.4% with metastatic disease.

**Table 1:** Characteristics of included patients

		Esophagus	Stomach
		2799	506
Sex (%)	Male	2207 (78.8)	305 (60.3)
	Female	592 (21.2)	201 (39.7)
Age (mean (SD))		66.19 (8.76)	66.90 (10.42)
Weight (mean (SD))		81.69 (16.21)	75.68 (15.12)
WHO Performance status (%)	0	1417 (55.9)	261 (60.3)
	1	991 (39.1)	136 (31.4)
	2	105 (4.1)	26 (6.0)
	3	19 (0.7)	10 (2.3)
	4	2 (0.1)	0 (0.0)
Tumor morphology (%)	Adenocarcinoma	2239 (80.0)	504 (99.6)
	Squamous cell carcinoma	540 (19.3)	0 ( 0.0)
	Other or unknown	20 (0.7)	2 (0.4)
Differentiation grade (%)	G1	110 (3.9)	8 (1.6)
	G2	1117 (39.9)	113 (22.3)
	G3	958 (34.2)	260 (51.4)
	G4	5 (0.2)	1 (0.2)
	Unknown	609 (21.8)	124 (24.5)
Hemoglobin (mean (SD))		9.86 (17.98)	7.74 (1.41)
Creatinine (mean (SD))		85.11 (155.64)	80.43 (28.29)
Albumine (mean (SD))		41.07 (21.07)	39.42 (17.44)
LDH (mean (SD))		203.97 (128.74)	204.84 (111.48)
cT (%)	1	50 (1.8)	11 (2.2)
	2	701 (25.0)	103 (20.4)
	3	1814 (64.8)	261 (51.6)
	4A	47 (1.7)	43 (8.5)
	4B	44 (1.6)	27 (5.3)
	X	143 (5.1)	61 (12.1)
cN (%)	0	1023 (36.5)	277 (54.7)
	1	1015 (36.3)	117 (23.1)
	2	603 (21.5)	82 (16.2)
	3	127 (4.5)	17 (3.4)
	X	31 (1.1)	13 (2.6)
cM (%)		0 (0.0)	0 (0.0)
	0	2352 (84.0)	393 (77.7)
	1	447 (16.0)	113 (22.3)
Treatment (%)	Neoadjuvant chemoradiotherapy followed by resection	1317 (47.1)	66 (13.0)
	Neoadjuvant chemoradiotherapy followed by resection followed by adjuvant nivolumab	74 (2.6)	0 (0.0)
	Neoadjuvant chemoradiotherapy not followed by resection	423 (15.1)	1 ( 0.2)
	Definitive chemoradiotherapy	245 ( 8.8)	6 (1.2)
	Neoadjuvant chemotherapy followed by resection	107 (3.8)	115 (22.7)
	Perioperative chemotherapy	95 (3.4)	124 (24.5)
	Endoscopic resection	23 (0.8)	5 (1.0)
	Resection	50 (1.8)	62 (12.3)
	Radiotherapy	94 (3.4)	6 (1.2)
	Systemic therapy	343 (12.3)	105 (20.8)
	Best supportive care	24 (0.9)	14 (2.8)
	Unknown or other	4 (0.1)	2 (0.4)

### Risk prediction model

**Fig. 2:**
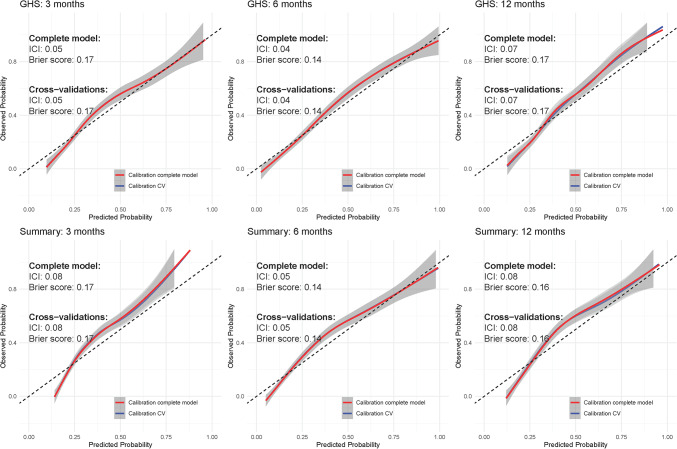
Calibration of the risk-prediction models for the Summary Score and GHS at 3, 6, and 12 months. The ICI and brier scores of the complete model and the average metrics of the cross-validated models are reported on the upper-right corner of each subfigure

**Fig. 3:**
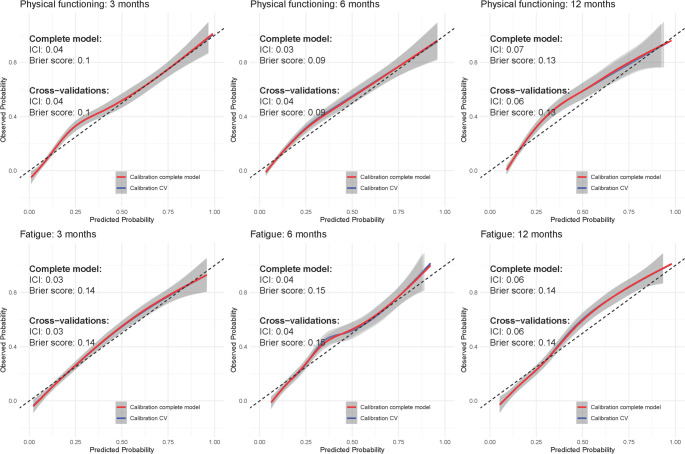
Feature importance of the GHS and Summary Score risk-prediction models. The list of displayed featured was truncated to show the top-10 most important features. Larger values of the absolute standardized coefficients correspond to higher feature importance

In total, 48 prediction models were developed for risk prediction of a significant deterioration at 3, 6 and 12 months after treatment onset (Supplementary Figs. 1, 2, 3 and Supplementary Table 6) for all patients with gastroesophageal cancer. The risk prediction models for the GHS and Summary Score can be found in Fig. 2. For the GHS models, the ICI varied between 0.04 and 0.07, the AUC was equal to 0.79 and the Brier score 0.14–0.17. The internal cross-validation showed identical estimates compared to the complete model (Fig. 2). The GHS baseline feature was the most important feature in predicting the risk of a significant deterioration across all time horizons (Fig. 3). For the 12-month model, the GHS baseline was the only feature that remained in the model after feature selection.

For the models predicting significant deteriorations in the Summary Score across all timepoints, the ICI of the complete model ranged between 0.05 and 0.08, the AUC was equal to 0.79 and the Brier score between 0.14 and 0.17. Results from internal cross-validations showed nearly identical estimates as the complete model. The most important features predicting the risk of a significant deterioration of the Summary Score were Social functioning, Constipation, and Appetite loss for the 3, 6 and 12 months models, respectively (Fig. 3).

**Fig. 4:**
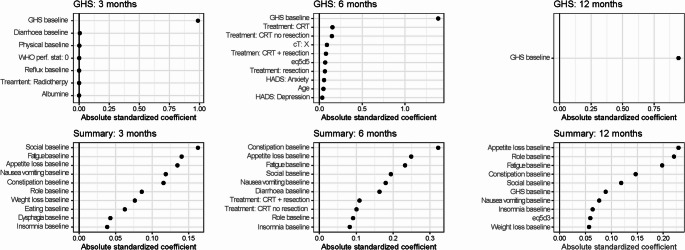
Calibration of the risk-prediction models for Fatigue and Physical functioning at 3, 6, and 12 months. The ICI and brier scores of the complete model and the average metrics of the cross-validated models are reported on the upper-right corner of each subfigure

**Fig. 5:**
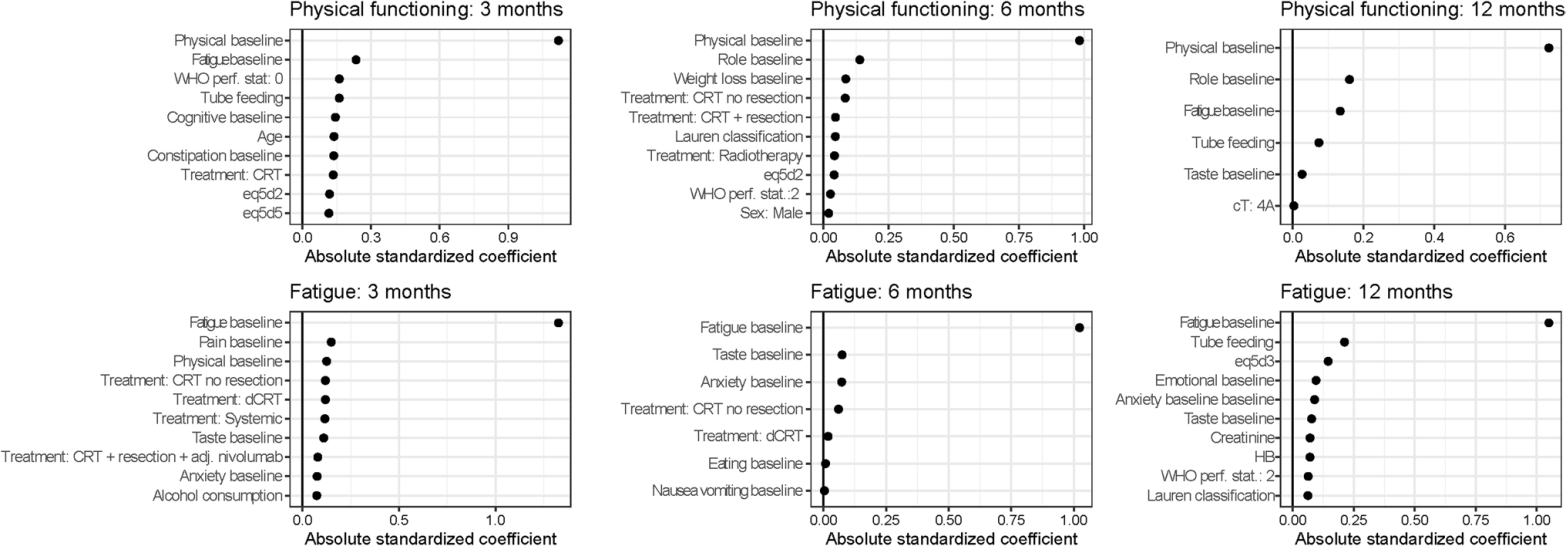
Feature importance of the Physical functioning and Fatigue risk-prediction models. The list of displayed featured was truncated to show the top-10 most important features. Larger values of the absolute standardized coefficients correspond to higher feature importance

Based on visual inspection of the calibration curves, we identified two outcomes that showed particularly good calibration: physical functioning and fatigue across all time horizons (Fig. 4). Results of all other outcomes are shown in Supplementary Figs. 1–3 and Supplementary Table 6. Note: As per style we don't provide a link for supplememntary material. Generally, the other outcomes demonstrated suboptimal calibration and are likely not useable for clinical practice. For Physical functioning the ICI varied between 0.03 and 0.07, the AUC was equal to 0.87 and the Brier score varied between 0.09 and 0.13. For Fatigue, the ICI varied between 0.03 and 0.06, the AUC varied between 0.83 and 0.84 and the Brier score ranges from 0.14 to 0.15 across all time horizons. For both Physical functioning and Fatigue the results from the internal cross-validation were nearly identical to the complete model. Both for Physical functioning and Fatigue the most important features were the Physical functioning and Fatigue baseline questionnaires (Fig. 5).

### Sequential score model

**Fig. 6:**
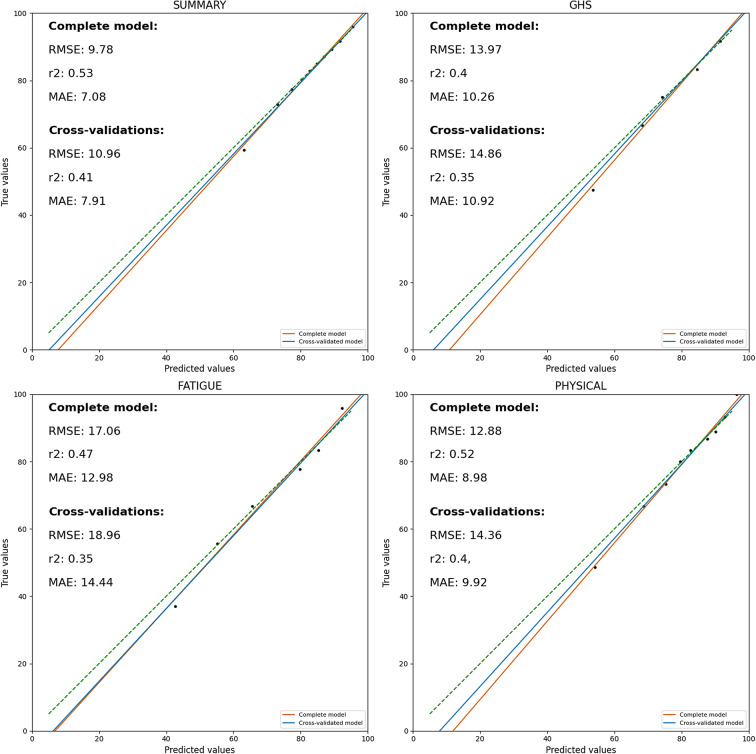
Calibration of the sequential score prediction models for the Summary Score, GHS, Fatigue and Physical functioning. The RMSE, r2 and MAE of the complete model and the average metrics of the cross-validated models are reported on the upper-left corner of each subfigure

The results of the internal external cross-validations and full models of Summary, GHS, Physical functioning and Fatigue are presented in Fig. 6.

**Fig. 7:**
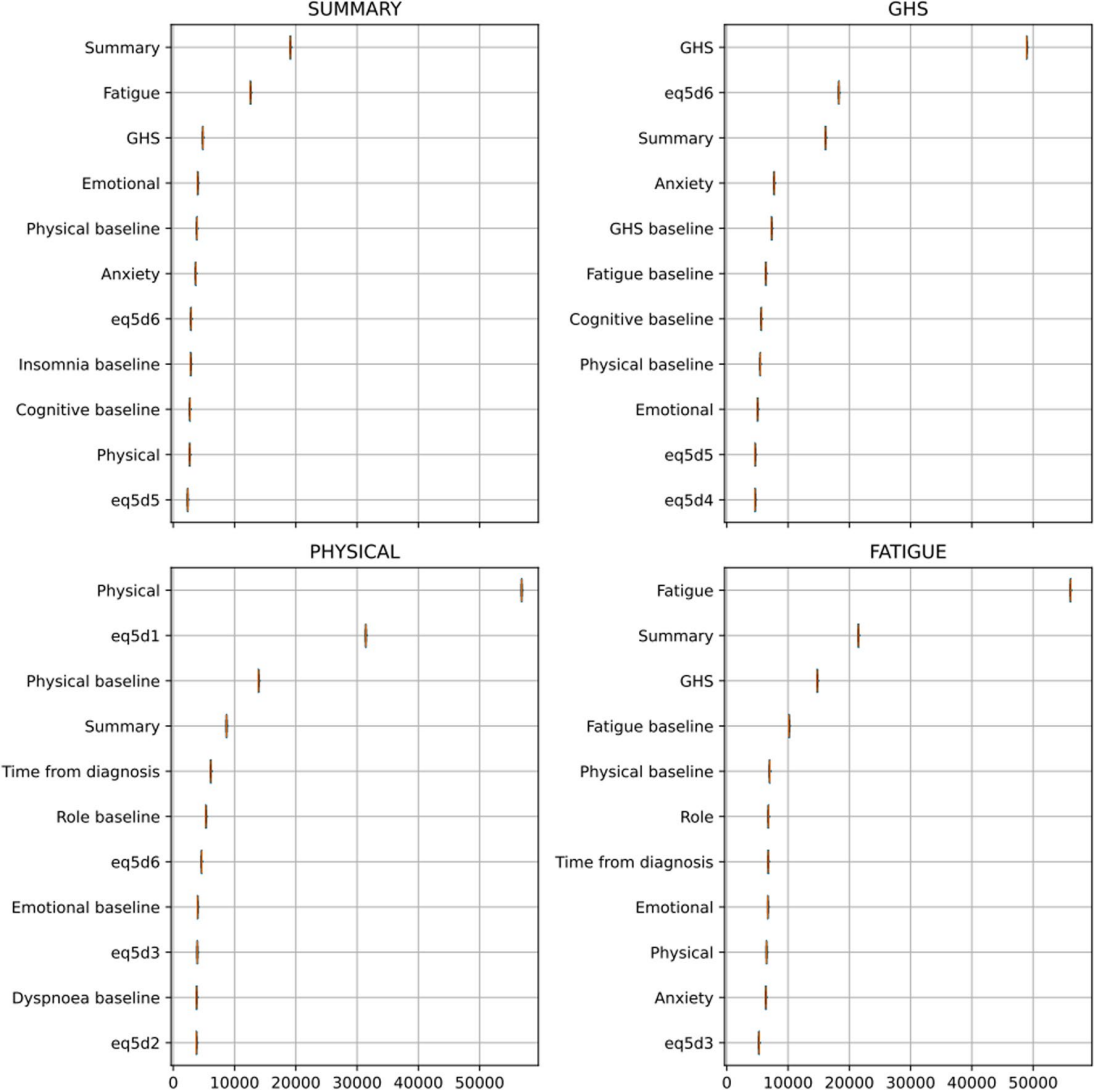
Feature importance of the sequential score models for the Summary Score, GHS, Physical function and Fatigue

For the summary score, the RMSE was 9.78, the mean absolute error (MEA) was 7.08 and the r2 was 0.53. Across cross-validations the mean RMSE was 11.0, MAE was 7.9 and r2 was 0.41. The most important features in terms of relative importance were Summary score at the previous questionnaire, Fatigue and Physical Functioning at baseline (Fig. 7). For the GHS, the RMSE was 13.97, the MAE was 10.26 and the r2 was 0.40. Across cross-validations the mean RMSE was 14.9, MAE was 11.0 and r2 was 0.35. GHS score at the previous questionnaire, Eq. 5D6 and the summary score were the most important features in terms of relative importance (Fig. 7).

Based on the visual inspection of the calibration curves, it can also be observed that, in line with the risk-prediction model, physical functioning and fatigue demonstrated good calibration (Fig. 6). For physical functioning, the RMSE was 12.88, the MAE was 8.98 and the r2 was 0.52. Across cross-validations the mean RMSE was 14.3, MAE was 9.9 and r2 was 0.40. The most important features in terms of relative importance were the Physical functioning score at the previous questionnaire, Eq. 5D1 and Physical functioning at baseline (Fig. 7). For the Fatigue score, the RMSE was 17.06, the MAE was 12.98 and the r2 was 0.47. Across cross-validations the mean RMSE was 19.0, MAE was 14.4 and r2 was 0.35. Fatigue score at the previous questionnaire, Summary and Role functioning showed the largest relative feature importance (Fig. 7).

In Supplementary Fig. 4, the calibration plots for all 16 outcomes, based on XGboost trained on the full dataset, are shown. These plots reflect the results based on the RMSE, MAE and r2 reported at the beginning of the section. Summary, Cognitive, Emotional, Financial and Physical score models appear to be well calibrated, especially for higher scores where the largest part of the outcome distribution lies. Nausea-vomiting, Dyspnoea, Insomnia, Constipation and Appetite loss did not show a good calibration, confirming previous results based on cross-validations. The results of the internal cross-validation for each of the 16 outcomes can be seen in Supplementary Fig. 5A–C. For the sequential score model, XGBoost outperformed Elastic net in terms of RMSE, MAE and r2 for all sixteen outcomes.

## Discussion

In this study we have presented a large number of models to predict health-related quality of life after treatment at primary diagnosis. Using two methodically different approaches we showed that we could successfully predict global health-related quality of life, as represented by the GHS and Summary Score, as well as physical functioning and fatigue for patients with gastroesophageal cancer.

### Model performance

Both the risk-prediction and sequential score models demonstrated good predictive performance for Summary Score, GHS, Physical Functioning, and Fatigue. Additionally, the sequential model showed strong performance for Cognitive, Emotional, and Financial difficulties. In general, reported AUC were good to excellent AUC, ranging between 0.78 (emotional functioning) to 0.98 (diarrhoea). Brier scores indicated that the risk-prediction models outperformed random chance, with good calibration and agreement between observed and predicted probabilities. Prediction quality was consistent across time horizons (3 months to 1 year post-treatment).

The sequential model also showed good calibration, explaining up to 40% of the variance (r^2^), but had a mean absolute error of 10–15 points, which corresponds to clinically significant changes in health-related quality of life. Despite good calibration, reducing this error may require modeling temporal variations throughout the patient journey, with future research focusing on integrating history from both questionnaires and clinical variables, and identifying additional predictive factors.

Results from internal validation of the risk-prediction model and the sequential score modeled indicated that there was relatively low overfitting of the models. The risk-prediction models in particular had model performance estimates of the complete model that were very similar to the estimates from internal cross-validations. This is likely attributed to the elastic net modeling, which can handle a large number of (collinear) variables without overfitting [26]. For the sequential score model, the complete model showed a slightly better performance than the estimates of the internal cross-validations. This difference, in the order of 5% for MAE, indicates that there might be some low overfitting, especially for the worst performing models. An explanation for this could lie on the tree-based nature of XGBoost, that is designed to improve incrementally performance.

### Predictive features

Across all models we observed that the most common and predictive features were the baseline questionnaires or the previous questionnaires for the risk-prediction model and sequential score model, respectively. Other predictive factors that were observed using both methodologies included EORTC QLQ-C30 and OG25 baseline questionnaires, the Hospital Anxiety and Depression scale and the Eq. 5D questionnaire. However, there were also a notable number of differences in the selected features in the risk prediction models and the sequential score models. Clinical variables such as cT, cN, age, performance status, hemoglobin, creatinine and treatment, were selected in a number of risk-prediction models, but none of the clinical variables except for treatment were selected in the sequential score models. This may indicate that such clinical variables may be predictive of the risk of a significant deterioration in health-related quality of life, but do not predict the score itself. A reason for this is that the clinical variables are measured at baseline only and not updated when the following questionnaires are filled in, making them less predictive in the short term for the sequential score.

### Clinical implications

Health-related quality of life is an essential aspect to consider when treating patients or improving patient care. To ensure a comprehensive perspective, focusing solely on one EORTC QLQ-C30 outcome may not be sufficient for informing patients and to further our understanding of prediction in this field. For this reason, we explored which outcomes could be predicted with adequate performance. Predicting multiple EORTC QLQ-C30 outcomes can help clinicians anticipate patient-reported challenges and tailor interventions proactively, rather than reactively. These predictions may also support shared decision-making, enabling patients to better understand potential trajectories of their quality of life and treatment impact. Furthermore, identifying outcomes with strong predictive performance provides a foundation for personalized care planning. By clarifying which outcomes are most predictable, we highlight where predictive tools can realistically add value in shared decision making, while acknowledging limitations for outcomes with lower predictive accuracy. In this light, we found that prediction of general health-related quality of life and outcomes on patients’ physical functioning and fatigue, could contribute meaningfully in clinical practice.

Since both global health-related quality of life and physical functioning and fatigue could be modeled and predicted with two different methodical approaches, this provides further evidence that the construct of health-related quality of life can be successfully predicted with a combination of clinical information such as personal, tumor and treatment related factors and baseline health-related quality of life factors. However, unlike results from health-related quality of life risk-prediction among colorectal cancer patients, our risk-prediction model did not find good predictive performance across all EORTC QLQ-C30 outcomes. It may be the case that for these outcomes, despite the comprehensive predictor set, we did not have the right predictors. The models predicting the Summary Score, GHS, Physical functioning, and Fatigue, seem to have adequate performance metrics to be used in clinical practice as the agreement between predicted and observed probabilities of a significant deterioration was very close. 

We are the first to predict a sequence of health-related quality of life scores. While this needs improvement in terms of reducing prediction error, the methodology looks very promising for future application. A sequential prediction model such as the one we have developed exhibits several advantageous characteristics compared to conventional risk-prediction models. One primary advantage is its ability to incorporate time as a continuous predictor. Unlike the risk-prediction model that typically predicts risk at fixed time intervals (e.g. three, six and twelve months post start of treatment), our sequential model allows prediction at any desired time point. This flexibility enhances its utility by enabling predictions at multiple time points of interest. With our sequential model, we can effectively model the trajectory of health-related quality of life by predicting and plotting the predicted health-related quality of life score across various time intervals. This capability is particularly valuable for patients as it provides a personalized prediction of their expected health-related quality of life trajectory throughout and following treatment.

Our models consistently highlighted baseline and previous health-related quality of life as the most informative predictors, whereas clinical variables such as tumor stage, performance status, or laboratory values contributed less once questionnaire data were available. This finding suggests that patient-reported outcomes may provide more actionable insights for anticipating future quality of life than routine clinical characteristics. While the primary aim of our work was methodological, these results may offer clinicians a first indication of which information is relevant when considering patient trajectories.

### Considerations

It is important to consider some limitations of this study. First of all, due to the observational nature of the data, the models cannot be used to select a treatment which has the lowest impact on health-related quality of life. Such a prediction would be a counterfactual prediction and is currently impossible with the published models. Second, the data that was selected for fitting the models should be extended to exploit their full potential. The sequential score prediction model predicts the score of the next questionnaire based on the last questionnaires and the clinical variables at baseline, but it would be recommendable to collect clinical variables every time a new questionnaire is filled in. Moreover, in the POCOP data, there was a disproportional amount of patients diagnosed with non-metastatic cancer as observed in the population. However, despite this discrepancy, an earlier study has shown that when accounting for treatment, as we have done in this study, it has very little effect on the real-world representativeness of findings obtained from POCOP data [27]. During the data preparation process, we decided to select only patient who had filled in the baseline questionnaire prior to treatment onset or less than 14 days after treatment onset, to align the patients at the same crucial time during their care trajectories. Finally, another important consideration in our study is the relatively large number of models estimated in relation to the sample size and number of predictors. While elastic net regression was used to reduce the risk of overfitting and to address collinearity, the development of multiple models may still increase the likelihood that some appear to perform well by chance. For this reason, the findings should be viewed primarily as methodological rather than definitive clinical results, and future external validation will be important to confirm the robustness of selected models.

These limitations notwithstanding, we have demonstrated two methodologically sound approaches which can be used to model and predict health-related quality of life and could also be used in different cancer settings. Testing two approaches allowed for the triangulation of evidence and given our findings that two approaches can both predict health-related quality of life, underscores the conclusion that health-related quality of life can in-fact be predicted. With this knowledge, the challenge for researchers working in this field is to find and refine predictive factors. Finally, in the future, it would be valuable if the presented models could be externally validated on new patient cohorts from the POCOP study or on other external data from a similar population of patients.

## Conclusion

This study successfully demonstrated that health-related quality of life for patients with esophageal and gastric cancer can be predicted using both risk-prediction and sequential score models. The models performed in predicting global health-related quality of life, Physical functioning and Fatigue. Our study highlights the promise of sequential prediction models, which allow for continuous predictions over time, offering personalized insights into patients’ HRQoL trajectories during and after treatment. This dual-method approach underscores the robustness of HRQoL prediction and highlights the potential of HRQoL prediction for improving of patient information and care.

## Supplementary Information

Below is the link to the electronic supplementary material.

Supplementary Material 1
