# Supplementary material for: Predicting health-related quality of life for patients with gastroesophageal cancer
Source: Qual Life Res. 2026 Feb 3;35(3):61. doi: 10.1007/s11136-025-04097-5 (PMC12868049; doi:10.1007/s11136-025-04097-5)
Supplement: Supplementary file 1 — Supplementary Material 1 [file 11136_2025_4097_MOESM1_ESM.docx]

**Supplementary Table 1.** Variables used for the risk-prediction models and the sequential models and the percentage of missing values at baseline.

| Variable | Pecentage of missing values [%] |
| --- | --- |
| WHO Performance status | 10.35 |
| Age | 0 |
| Biological sex | 0 |
| Tumour orphology | 0 |
| Lauren classification | 18.52 |
| cT | 0.03 |
| cN | 0.03 |
| cM | 0.03 |
| Treatment | 0 |
| Hemoglobine | 4.21 |
| Creatinine | 4.84 |
| Lactic acid dehydrogenase (LDH) | 17.97 |
| Albumin | 27.69 |
| Differentation grade | 0 |
| Height | 8.08 |
| Weight | 7.897125567 |
| Tumour location | 0 |
| EORTC C30 GHS | 0.907715582 |
| EORTC C30 Fatigue | 0.605143722 |
| EORTC C30 Nausea/Vomiting | 0.665658094 |
| EORTC C30 Pain | 0.423600605 |
| EORTC C30 Dyspnoea | 0.756429652 |
| EORTC C30 Insomnia | 0.69591528 |
| EORTC C30 Appetite loss | 1.149773071 |
| EORTC C30 Constipation | 0.756429652 |
| EORTC C30 Diarrhoea | 0.756429652 |
| EORTC C30 Financial | 0.907715582 |
| EORTC C30 Physical functioning | 0.605143722 |
| EORTC C30 Role | 0.605143722 |
| EORTC C30 Emotional functioning | 0.69591528 |
| EORTC C30 Cognitive functioning | 0.69591528 |
| EORTC C30 Social functioning | 0.756429652 |
| EORTC OG25 Dysphagia | 1.059001513 |
| EORTC OG25 Eating | 1.936459909 |
| EORTC OG25 Reflux | 1.54311649 |
| EORTC OG25 Odynophagia | 1.966717095 |
| EORTC OG25 Pain dyscomfort | 1.694402421 |
| EORTC OG25 Anxiety | 0.968229955 |
| EORTC OG25 Eating/others | 2.027231467 |
| EORTC OG25 Dry mouth | 1.119515885 |
| EORTC OG25 Taste | 1.512859304 |
| EORTC OG25 Body image | 1.059001513 |
| EORTC OG25 Swallow saliva | 0.907715582 |
| EORTC OG25 Choking swallowing | 1.149773071 |
| EORTC OG25 Coughing | 1.270801815 |
| EORTC OG25 Talking | 1.210287443 |
| EORTC OG25 Weight loss | 1.119515885 |
| Education level | 1.54311649 |
| Married | 1.39183056 |
| EuroQoL eq5d1 | 0.756429652 |
| EuroQoL eq5d2 | 0.786686838 |
| EuroQoL eq5d3 | 0.726172466 |
| EuroQoL eq5d4 | 0.635400908 |
| EuroQoL eq5d5 | 0.69591528 |
| EuroQoL eq5d6 | 1.39183056 |
| HADS depression | 1.996974281 |
| HADS happy | 2.08774584 |
| Time from treatment onset | 0.968229955 |

**Supplementary Table 2.** Cut-off scores used in the risk-prediction model to determine a clinically meaningful deterioration in health-related quality of life.

| Scale | Cut-off |
| --- | --- |
| GHS | 5 |
| EORTC C30: Fatigue | 5 |
| EORTC C30: Nausea vomiting | 5 |
| EORTC C30: Pain | 3 |
| EORTC C30: Dyspnea | 5 |
| EORTC C30: Insomnia | 2 |
| EORTC C30: Appetite loss | 2 |
| EORTC C30: Constipation | 5 |
| EORTC C30: Diarrhoea | 5 |
| EORTC C30: Financial difficulty | 2 |
| EORTC C30: Physical functioning | 5 |
| EORTC C30: Role functioning | 7 |
| EORTC C30: Emotional functioning | 3 |
| EORTC C30: Cognitive functioning | 1 |
| EORTC C30: Social functioning | 6 |
| EORTC C30: Summary score | 4 |

**Supplementary Table 3.** Prevalences of clinically significant deterioration**s** at 3 months, 6 months and 12 months.

|  | Outcome | Prevalence (%) |
| --- | --- | --- |
|  |  |  |
| 3 months | EORTC_C30_GHS_GHS | 33,1 |
|  | EORTC_C30_SYMPTOM_FATIGUE | 26,6 |
|  | EORTC_C30_SYMPTOM_NAUSEA_VOMITING | 27,5 |
|  | EORTC_C30_SYMPTOM_PAIN | 29,3 |
|  | EORTC_C30_SYMPTOM_DYSPNOEA | 10,1 |
|  | EORTC_C30_SYMPTOM_INSOMNIA | 24,4 |
|  | EORTC_C30_SYMPTOM_APPETITE_LOSS | 25,8 |
|  | EORTC_C30_SYMPTOM_CONSTIPATION | 23,0 |
|  | EORTC_C30_SYMPTOM_DIARRHOEA | 12,0 |
|  | EORTC_C30_SYMPTOM_FINANCIAL | 7,3 |
|  | EORTC_C30_FUNCTION_PHYSICAL | 16,8 |
|  | EORTC_C30_FUNCTION_ROLE | 19,0 |
|  | EORTC_C30_FUNCTION_EMOTIONAL | 45,4 |
|  | EORTC_C30_FUNCTION_COGNITIVE | 17,9 |
|  | EORTC_C30_FUNCTION_SOCIAL | 21,3 |
|  | EORTC_C30_SUMMARY_SUMMARY | 28,2 |
|  |  |  |
| 6 months | EORTC_C30_GHS_GHS | 30,4 |
|  | EORTC_C30_SYMPTOM_FATIGUE | 26,0 |
|  | EORTC_C30_SYMPTOM_NAUSEA_VOMITING | 24,3 |
|  | EORTC_C30_SYMPTOM_PAIN | 28,4 |
|  | EORTC_C30_SYMPTOM_DYSPNOEA | 10,6 |
|  | EORTC_C30_SYMPTOM_INSOMNIA | 26,2 |
|  | EORTC_C30_SYMPTOM_APPETITE_LOSS | 20,9 |
|  | EORTC_C30_SYMPTOM_CONSTIPATION | 25,5 |
|  | EORTC_C30_SYMPTOM_DIARRHOEA | 10,8 |
|  | EORTC_C30_SYMPTOM_FINANCIAL | 6,5 |
|  | EORTC_C30_FUNCTION_PHYSICAL | 15,0 |
|  | EORTC_C30_FUNCTION_ROLE | 18,8 |
|  | EORTC_C30_FUNCTION_EMOTIONAL | 50,1 |
|  | EORTC_C30_FUNCTION_COGNITIVE | 17,3 |
|  | EORTC_C30_FUNCTION_SOCIAL | 21,1 |
|  | EORTC_C30_SUMMARY_SUMMARY | 25,1 |
|  |  |  |
| 12 months | EORTC_C30_GHS_GHS | 37,4 |
|  | EORTC_C30_SYMPTOM_FATIGUE | 31,0 |
|  | EORTC_C30_SYMPTOM_NAUSEA_VOMITING | 23,6 |
|  | EORTC_C30_SYMPTOM_PAIN | 29,5 |
|  | EORTC_C30_SYMPTOM_DYSPNOEA | 11,8 |
|  | EORTC_C30_SYMPTOM_INSOMNIA | 26,4 |
|  | EORTC_C30_SYMPTOM_APPETITE_LOSS | 25,6 |
|  | EORTC_C30_SYMPTOM_CONSTIPATION | 23,4 |
|  | EORTC_C30_SYMPTOM_DIARRHOEA | 11,1 |
|  | EORTC_C30_SYMPTOM_FINANCIAL | 7,0 |
|  | EORTC_C30_FUNCTION_PHYSICAL | 22,3 |
|  | EORTC_C30_FUNCTION_ROLE | 23,4 |
|  | EORTC_C30_FUNCTION_EMOTIONAL | 51,7 |
|  | EORTC_C30_FUNCTION_COGNITIVE | 18,5 |
|  | EORTC_C30_FUNCTION_SOCIAL | 28,9 |
|  | EORTC_C30_SUMMARY_SUMMARY | 32,5 |

**Supplementary Table 4.** Elastic net parameters (Alpha and Lambda) for all models across all timepoints.

|  | Outcome | Alpha | Lambda |
| --- | --- | --- | --- |
| 3 months | COGNITIVE | 0,729 | 0,033 |
|  | EMOTIONAL | 0,675 | 0,032 |
|  | PHYSICAL | 0,781 | 0,008 |
|  | ROLE | 0,539 | 0,024 |
|  | *SOCIAL* | 0,846 | 0,035 |
|  | GHS | 0,890 | 0,029 |
|  | SUMMARY | 0,241 | 0,148 |
|  | APPETITE LOSS | 0,729 | 0,033 |
|  | CONSTIPATION | 0,846 | 0,035 |
|  | DIARRHOEA | 0,928 | 0,016 |
|  | DYSPNOEA | 0,639 | 0,070 |
|  | FATIGUE | 0,954 | 0,011 |
|  | FINANCIAL | 0,714 | 0,009 |
|  | INSOMNIA | 0,675 | 0,032 |
|  | NAUSEA*/*VOMITING | 0,594 | 0,044 |
|  | PAIN | 0,956 | 0,028 |
|  |  |  |  |
| 6 months | COGNITIVE | 0,868 | 0,025 |
|  | EMOTIONAL | 0,947 | 0,016 |
|  | PHYSICAL | 0,880 | 0,021 |
|  | ROLE | 0,527 | 0,043 |
|  | *SOCIAL* | 0,985 | 0,023 |
|  | GHS | 0,947 | 0,016 |
|  | SUMMARY | 0,495 | 0,032 |
|  | APPETITE LOSS | 0,868 | 0,025 |
|  | CONSTIPATION | 0,985 | 0,023 |
|  | DIARRHOEA | 0,947 | 0,016 |
|  | DYSPNOEA | 0,430 | 0,032 |
|  | FATIGUE | 0,968 | 0,030 |
|  | FINANCIAL | 0,962 | 0,032 |
|  | INSOMNIA | 0,693 | 0,040 |
|  | NAUSEA*/*VOMITING | 0,957 | 0,029 |
|  | PAIN | 0,962 | 0,012 |
|  |  |  |  |
| 12 months | COGNITIVE | 0,959 | 0,016 |
|  | EMOTIONAL | 0,989 | 0,006 |
|  | PHYSICAL | 0,508 | 0,059 |
|  | ROLE | 0,938 | 0,011 |
|  | *SOCIAL* | 0,729 | 0,017 |
|  | GHS | 0,898 | 0,061 |
|  | SUMMARY | 0,231 | 0,118 |
|  | APPETITE LOSS | 0,959 | 0,016 |
|  | CONSTIPATION | 0,729 | 0,017 |
|  | DIARRHOEA | 0,898 | 0,061 |
|  | DYSPNOEA | 0,673 | 0,022 |
|  | FATIGUE | 0,317 | 0,040 |
|  | FINANCIAL | 0,317 | 0,040 |
|  | INSOMNIA | 0,898 | 0,061 |
|  | NAUSEA*/*VOMITING | 0,957 | 0,028 |
|  | PAIN | 0,935 | 0,058 |

**Supplementary Table 5.** *XGboost parameters* for all models.

| Outcome | colsample_bytree | gamma | learning_rate | max_depth | min_child_weight | n_estimators | reg_alpha | reg_lambda | *subsample* |
| --- | --- | --- | --- | --- | --- | --- | --- | --- | --- |
| COGNITIVE | *0.58* | *5.41* | *0.03* | *4* | *8.0* | *300* | *27.09* | *2.69* | *0.67* |
| EMOTIONAL | *0.86* | *5.08* | *0.04* | *3* | *5.0* | *200* | *31.80* | *1.57* | *0.52* |
| PHYSICAL | *0.90* | *7.14* | *0.06* | *3* | *7.0* | *250* | *28.62* | *2.89* | *0.91* |
| ROLE | *0.64* | *1.23* | *0.02* | *5* | *8.0* | *400* | *19.12* | *4.36* | *0.90* |
| *SOCIAL* | *0.66* | *4.42* | *0.04* | *4* | *7.0* | *200* | *13.51* | *1.31* | *0.83* |
| GHS | *0.91* | *5.20* | *0.02* | *3* | *10.0* | *450* | *48.12* | *1.65* | *0.52* |
| SUMMARY | *0.62* | *5.67* | *0.05* | *4* | *3.0* | *200* | *42.44* | *3.04* | *0.71* |
| APPETITE LOSS | *0.93* | *5.97* | *0.06* | *4* | *6.0* | *150* | *49.89* | *2.06* | *0.65* |
| CONSTIPATION | *0.81* | *3.51* | *0.12* | *3* | *1.0* | *100* | *44.30* | *4.32* | *0.61* |
| DIARRHOEA | *0.97* | *1.44* | *0.08* | *3* | *0.0* | *200* | *31.33* | *1.96* | *0.90* |
| DYSPNOEA | *0.90* | *2.23* | *0.08* | *3* | *3.0* | *250* | *1.25* | *4.24* | *0.64* |
| FATIGUE | *0.90* | *1.99* | *0.04* | *4* | *10.0* | *200* | *9.03* | *3.80* | *0.77* |
| FINANCIAL | 1.0 | 0.14 | 0.06 | 3 | 7.0 | 100 | 50.0 | 3.84 | 0.63 |
| INSOMNIA | 0.94 | 5.77 | 0.05 | 3 | 9.0 | 200 | 48.40 | 2.71 | 0.76 |
| NAUSEA*/*VOMITING | *0.91* | *7.70* | *0.05* | *3* | *8.0* | *300* | *21.61* | *0.66* | *0.68* |
| PAIN | *0.51* | *4.11* | *0.02* | *4* | *8.0* | *400* | *19.07* | *4.95* | *0.88* |

**Supplementary Table 6.** AUC of the risk-prediction models predicting the 3, 6 and 12 months risk of a significant deterioration. Results are reported averaged for the cross-validation (CV) and for the complete model trained on the whole dataset.

| Outcome | AUC 3 months CV | AUC 3 months Complete model | AUC 6 months CV | AUC 6 months Complete model | AUC 12 months CV | AUC 12 months Complete model |
| --- | --- | --- | --- | --- | --- | --- |
| COGNITIVE | 0.90 | 0.90 | 0.90 | 0.90 | 0.90 | 0.90 |
| EMOTIONAL | 0.80 | 0.78 | 0.78 | 0.78 | 0.78 | 0.78 |
| PHYSICAL | 0.87 | 0.87 | 0.87 | 0.87 | 0.87 | 0.87 |
| ROLE | 0.89 | 0.89 | 0.89 | 0.89 | 0.89 | 0.89 |
| *SOCIAL* | 0.87 | 0.87 | 0.87 | 0.87 | 0.87 | 0.87 |
| GHS | 0.79 | 0.79 | 0.79 | 0.79 | 0.79 | 0.79 |
| SUMMARY | 0.79 | 0.79 | 0.79 | 0.79 | 0.79 | 0.79 |
| APPETITE LOSS | 0.91 | 0.91 | 0.91 | 0.91 | 0.91 | 0.91 |
| CONSTIPATION | 0.93 | 0.93 | 0.93 | 0.93 | 0.93 | 0.93 |
| DIARRHOEA | 0.97 | 0.98 | 0.97 | 0.98 | 0.97 | 0.98 |
| DYSPNOEA | 0.91 | 0.91 | 0.91 | 0.91 | 0.91 | 0.91 |
| FATIGUE | 0.83 | 0.84 | 0.83 | 0.84 | 0.83 | 0.84 |
| FINANCIAL | 0.97 | 0.98 | 0.97 | 0.98 | 0.97 | 0.98 |
| INSOMNIA | 0.87 | 0.87 | 0.87 | 0.87 | 0.87 | 0.87 |
| NAUSEA*/*VOMITING | 0.94 | 0.94 | 0.94 | 0.94 | 0.94 | 0.94 |
| PAIN | 0.87 | 0.87 | 0.87 | 0.87 | 0.87 | 0.87 |


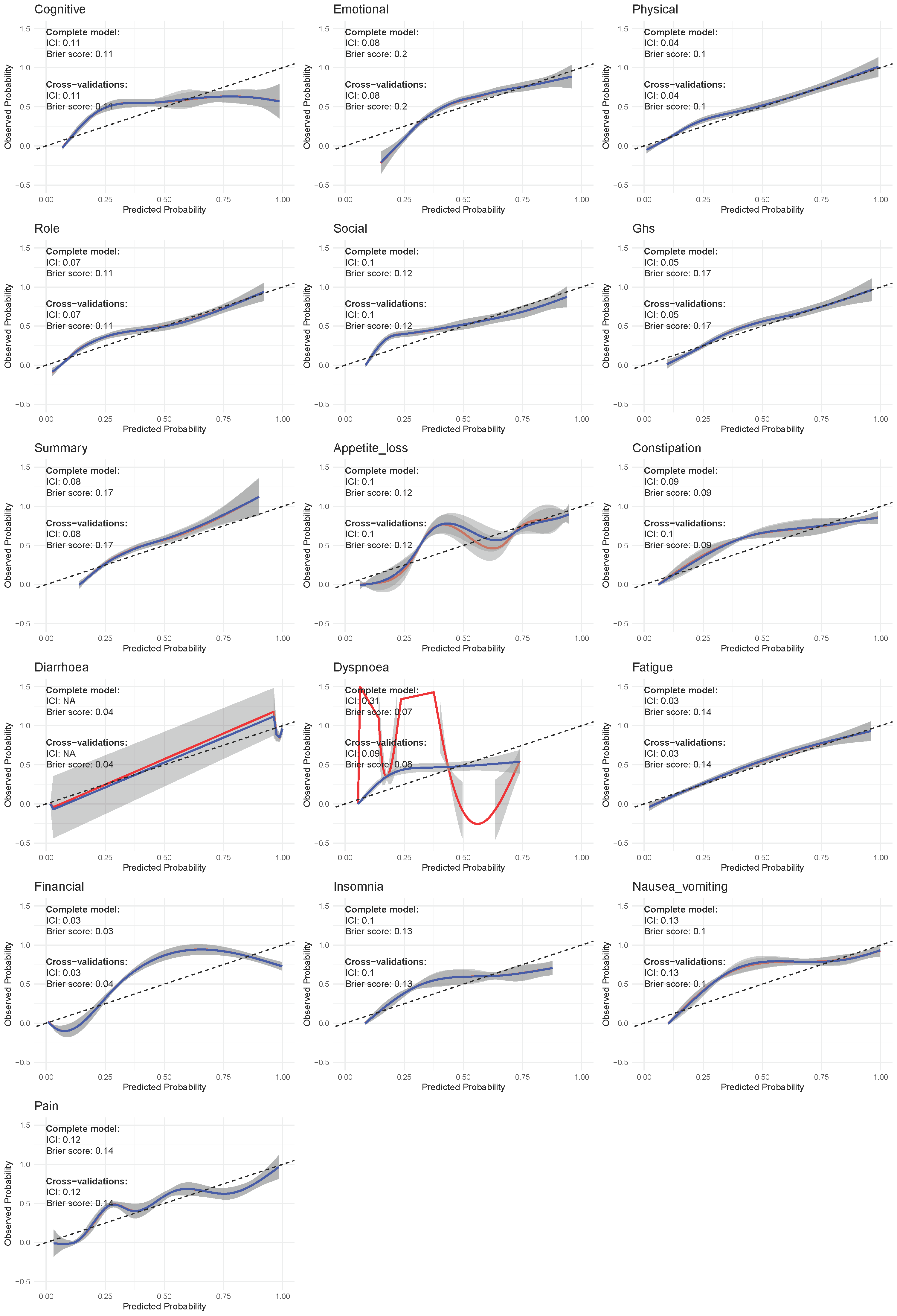


**Supplementary Figure 1.** Calibration of the risk-prediction models predicting the 3 months risk of a significant deterioration.


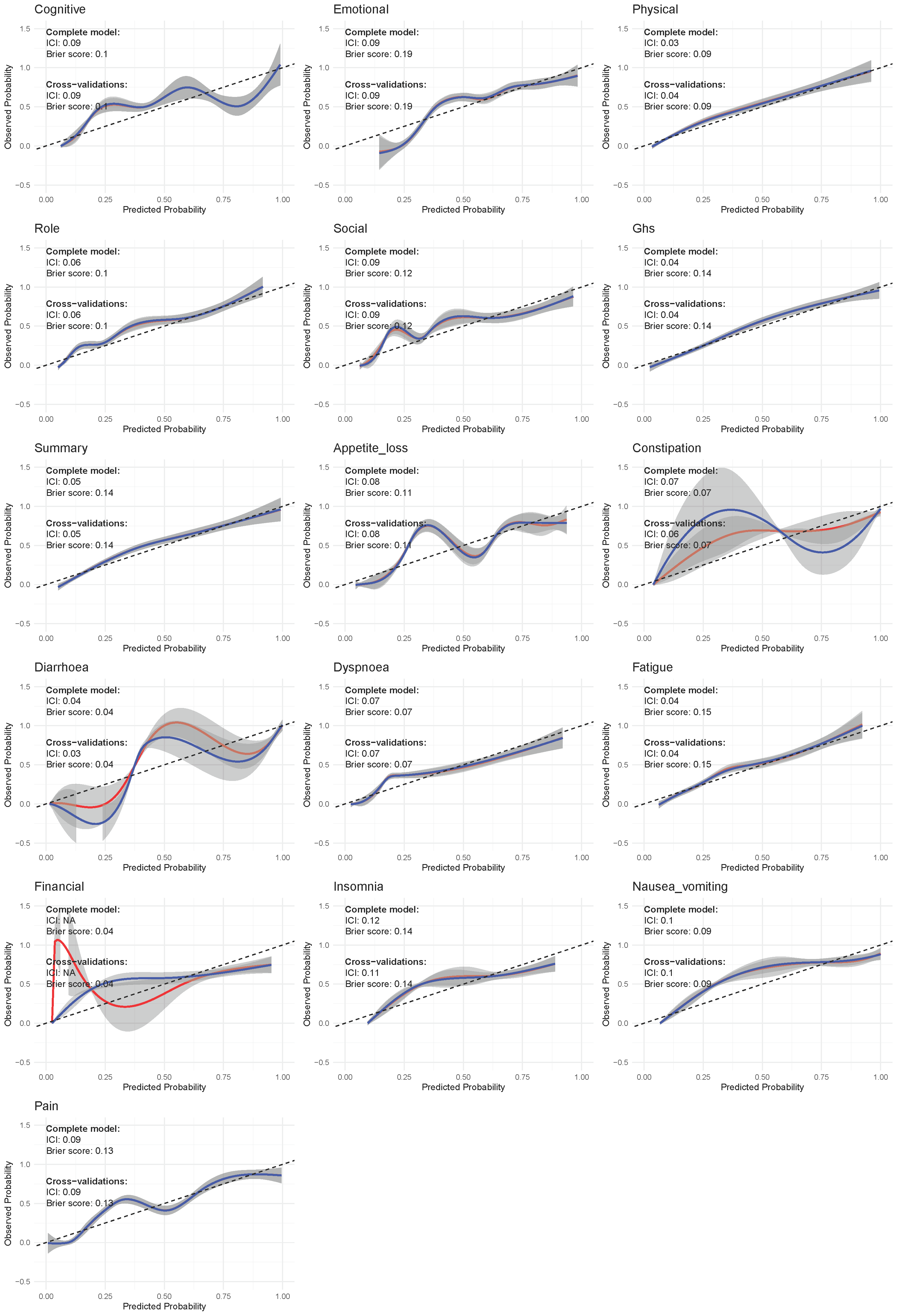


**Supplementary Figure 2.** Calibration of the risk-prediction models predicting the 6 months risk of a significant deterioration.


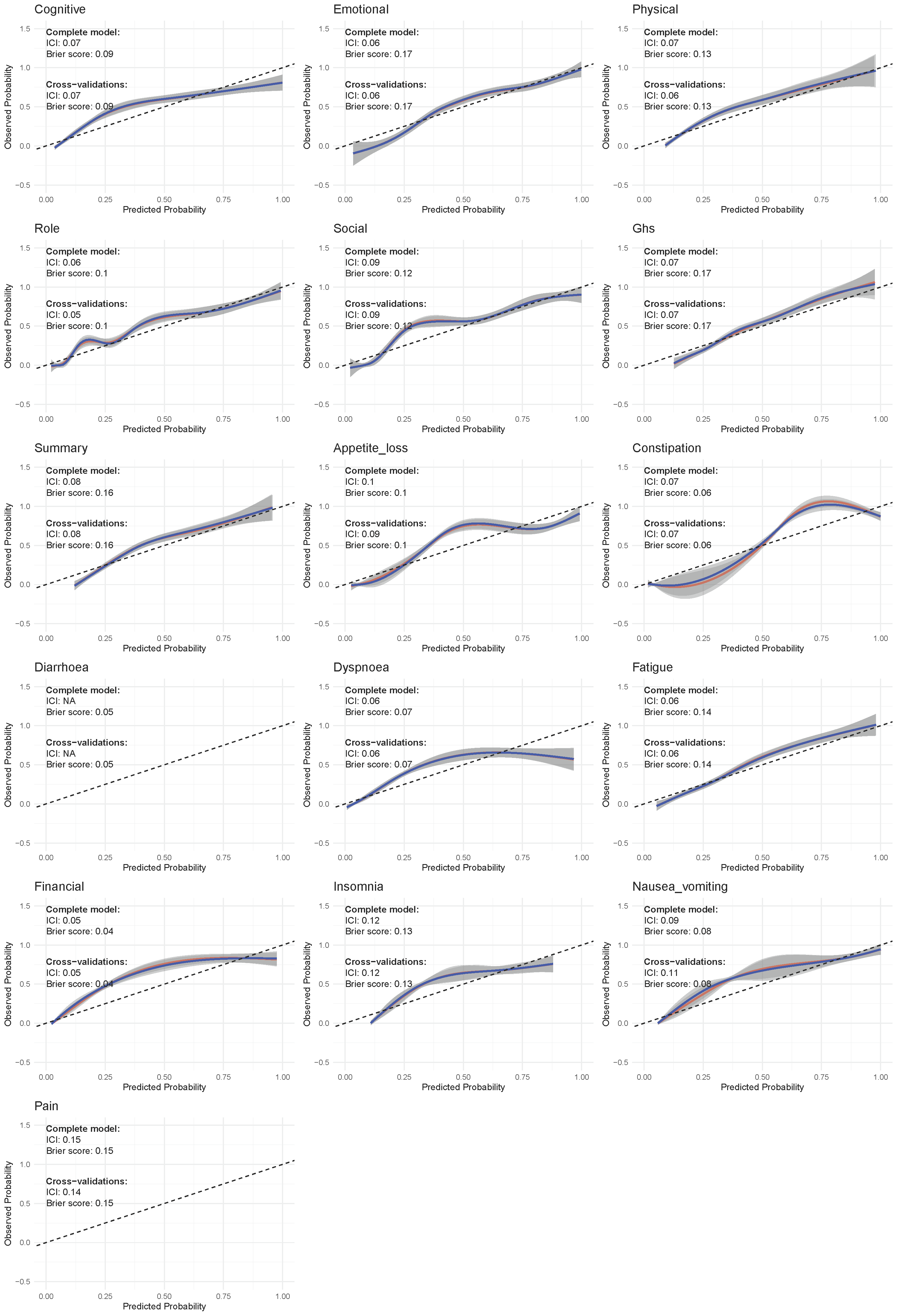


**Supplementary Figure 3.** Calibration of the risk-prediction models predicting the 12 months risk of a significant deterioration. Note: the model predicting Diarrhoea and Pain could not converge.


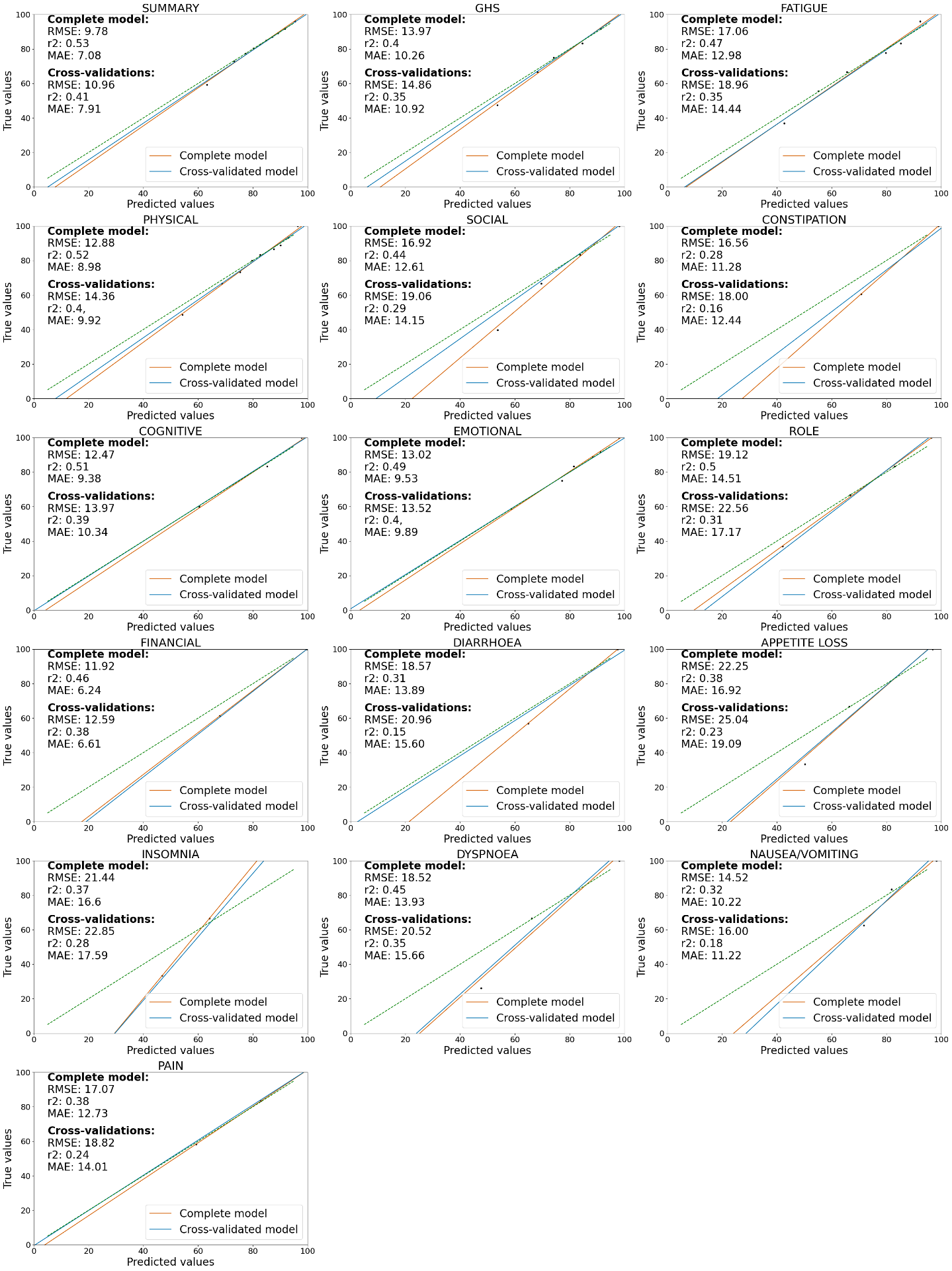


**Supplementary Figure 4.** Calibration of the sequential score prediction models for all 16 outcomes. The RMSE, r2 and MAE of the complete model and the average metrics of the cross-validated models are reported on the upper-right corner of each subfigure.


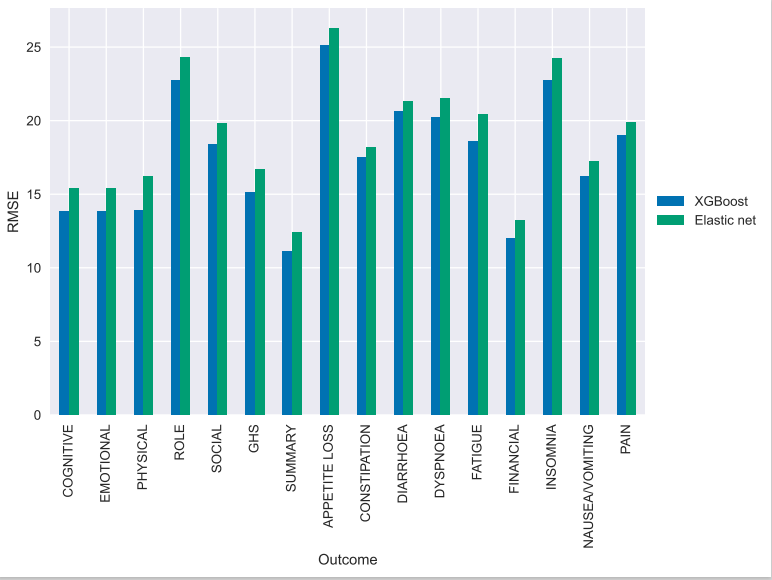


**Supplementary Figure 5A.** Root mean squared error (RMSE) of Elastic net and XGBoost models.


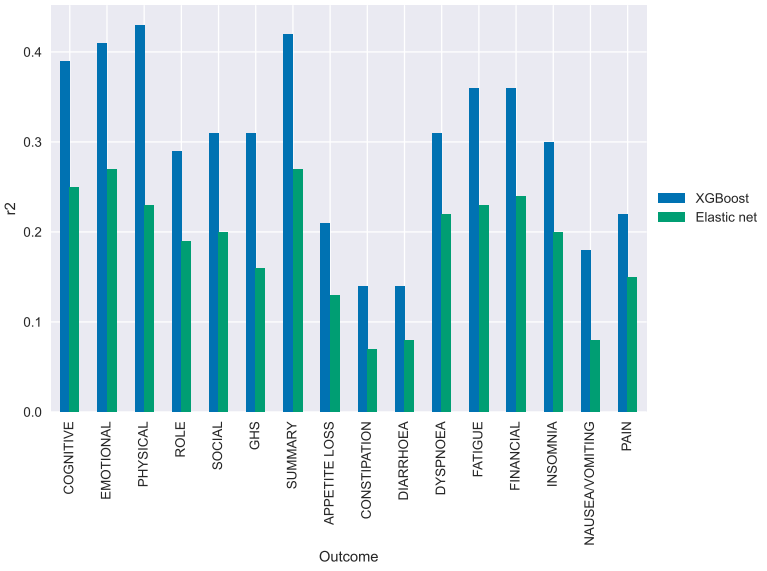


**Supplementary Figure 5B.** R2 of Elastic net and XGBoost models.


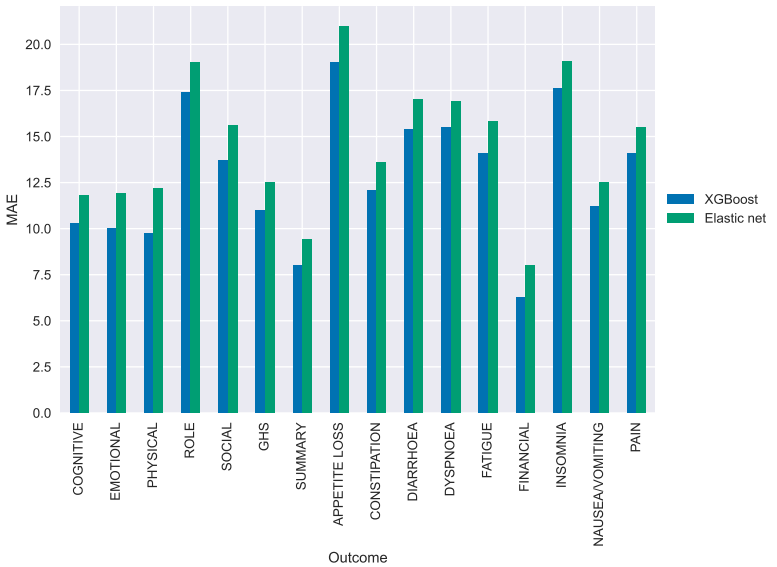


**Supplementary Figure 5C.** Mean absolute error (MEA) of Elastic net and XGBoost models.
